# Correlation of Platelet Indices in Patients With Type 2 Diabetes Mellitus and Associated Microvascular Complications: A Hospital-Based, Prospective, Case-Control Study

**DOI:** 10.7759/cureus.55959

**Published:** 2024-03-11

**Authors:** Parul Khanna, Surinder K Salwan, Aditya Sharma

**Affiliations:** 1 Department of General Medicine, Sri Guru Ram Das University of Health Sciences, Amritsar, IND; 2 Department of General Surgery, Institute of Medical Sciences, Banaras Hindu University, Varanasi, IND

**Keywords:** diabetic microvascular complications, endocrinology and diabetes, platelet indices, mean platelet volume(mpv), type 2 diabetes mellites

## Abstract

Background

Diabetic patients exhibit increased platelet activity. Insulin inhibits the activation of platelets. Therefore, a relative or absolute deficiency of insulin would increase platelet reactivity. The younger (larger) platelets are also more metabolically and enzymatically active.^ ^If detected early, microvascular complications could alert us regarding the possible macrovascular complications. Thus, the aims and objectives of the present study were to determine platelet indices in patients with type 2 diabetes mellitus with controls (non-diabetics) and to find an association of platelet indices with microvascular complications.

Material & methods

In this prospective case-control study conducted from 2021 to 2022 (2 years), a total number of 200 subjects were taken and were divided into two groups of 100 each, cases (I) and controls (II). The cases included patients of diabetes mellitus (DM) of a duration of more than 5 years, which were further divided into two groups of 50 each, IA and IB. Group IA consisted of patients with diabetes mellitus of a duration of more than five years with at least one microvascular complication and group IB was diabetics of more than five years duration without any microvascular complications, which includes diabetic retinopathy, diabetic nephropathy, and diabetic neuropathy. An automated cell counter (Thermo Fisher Scientific, Waltham, MA, US) provided hemoglobin values along with the platelet count and platelet indices, i.e. mean platelet volume (MPV), platelet large cell ratio (P-LCR), and platelet distribution width (PDW).

Results

The present study consisted of 200 subjects divided into 2 groups of 100 each, cases (I) and controls (II). The average MPV (9.4-12.3 femtolitre) in diabetics was 12.089±1.450 fL as compared to the controls where it was 9.464±1.424 fL with a statistically significant p-value of 0.001. PDW among the cases was 16.868±2.352 fL while in controls, it was 12.753±10.559 fL (p=0.001). The mean P-LCR was 34.975±8.056% among the cases, in comparison to the mean P-LCR among the controls, which was 26.031±7.004 (p=0.001). In this study, the MPV, PDW, and P-LCR were significantly raised in individuals having diabetes with microvascular complications when compared with patients without complications. The mean MPV in diabetics with complications was 12.5960±0.95660 fL and in those without complications was 11.5820±1.67609 fL (with a p-value of P = 2×10^−3^)which is statistically significant. Similar results were obtained in cases of PDW and P-LCR. The mean PDW in diabetics with complications was 17.1140±2.58228 fL and without complications was 15.6220±2.10532 fL ((with a p-value of P = 2×10^−3^)). The mean P-LCR in diabetics with microvascular complications was 35.408±3.5490% and without complications was 33.542±4.8694% (with a p-value of P = 3.1×10^−3^).

Conclusion

Based on the findings of the present study, there is a statistical correlation between type 2 diabetes and variations in platelet indices, resulting in the associated microvascular complications. Higher MPV, PDW, and P-LCR values suggest that these parameters are more reliable predictors of early vascular complications in individuals with type 2 diabetes mellitus and can be utilized as an easy-to-use, low-cost method. They are a readily available, economical, practical, noninvasive, and simple-to-understand approach for assessing platelet dysfunction, which in turn helps anticipate the existence of microvascular complications.

## Introduction

Diabetes mellitus (DM) is a metabolic syndrome characterized by chronic hyperglycemia [1,2]. Symptoms often include increased frequency of urination, thirst, and appetite. Untreated type 2 diabetes mellitus (T2DM) leads to a variety of complications [3-5]. Acute complications include diabetic ketoacidosis, hyperosmolar hyperglycemic state, or death [6]. Serious long-term complications include macrovascular complications (cardiovascular disease, cerebrovascular accident, foot ulcers) and microvascular complications (damage to the nerves leading to diabetic neuropathy, damage to the eyes causing diabetic retinopathy, and renal involvement known as diabetic nephropathy) [6,7]. Microvascular complications, such as retinal lesions, microalbuminuria, and proteinuria, have been described as factors predictive of cardiovascular and cerebrovascular morbidity and mortality among diabetic subjects [7]. Diabetic patients exhibit increased platelet activity by encouraging the glycation of platelet proteins and associated hyperglycemia increases platelet reactivity [5-7]. Both insulin resistance and insulin deficiency increase platelet reactivity. Insulin prevents platelet activation. Therefore, an increase in platelet reactivity would result from an absolute or relative insulin deficiency [4,5].

Additionally, the up-regulation of glycoproteins (Ib and IIb/IIIa) and P2Y12 signaling, which are important processes underpinning atherothrombotic risk in T1DM and T2DM, are caused by hyperglycemia. Possible mechanisms by which elevated glucose promotes vascular abnormalities include disturbances in polyol pathways, activation of protein kinase C, and formation of advanced glycation end products. An increase in thromboxane A2, serotonin, and thromboglobulin, or a decrease in prostacyclin synthesis are associated with platelet hyperactivity. Osmotic edema brought on by elevated blood sugar and possibly owing to a shortened platelet lifespan in diabetic individuals is one potential explanation of increased MPV in DM [7,8].

The mean platelet volume (MPV), platelet distribution width (PDW), and platelet large cell ratio (P-LCR) are three different platelet indicators that show how crucial platelets are to maintaining the integrity of normal homeostasis [9]. It is seen that larger platelets have a higher number of dense granules, making them more potent and thrombogenic [10]. Microvascular complications, such as retinal lesions, microalbuminuria, and proteinuria, have been described as factors predictive of cardiovascular and cerebrovascular morbidity and mortality among diabetic subjects.

## Materials and methods

Aim and objectives 

The aim and objectives of the present study were to determine platelet indices in patients with type 2 diabetes mellitus and healthy controls, to find an association of platelet indices with type 2 diabetes mellitus, and to find an association of platelet indices with microvascular complications in patients of type 2 diabetes mellitus.

Materials

The present cross-sectional study was undertaken from 2021-2022 on 200 subjects, ages 18 years or above, visiting the outpatient or inpatient department of medicine, Sri Guru Ram Das Institute of Medical Sciences & Research (SGRDIMSR), Amritsar. They were further divided into two groups of 100 each.

The study was done after obtaining ethical clearance from the ethical committee of SGRDIMSR (approval number ANT/68/2021.

Table 1 shows the group distribution. 

**Table 1:** Group distribution between patients and controls

Group I (Cases)	Group II (Controls)
Diabetic patients diagnosed according to American Diabetes Association (ADA) criteria with a duration of more than five years. These cases were further subdivided into two groups of 50 each: I A - Subjects with at least one microvascular complication. I B - Subjects without any microvascular complications.	Age and sex-matched healthy controls

Inclusion and exclusion criteria 

Table 2 shows the inclusion and exclusion criteria for the present study.

**Table 2:** Inclusion and exclusion criteria for the present study HbA1C: glycated hemoglobin

Inclusion Criteria	Exclusion Criteria
Diabetic patients > 18 years diagnosed according to American Diabetes Association (ADA) criteria for > 5 years with at least one microvascular complication in the form of diabetic neuropathy, diabetic retinopathy, and diabetic nephropathy.	Abnormal platelet count (<1.0 and >4.0 Lakhs/cumm), pregnant females, and patients with any diagnosed malignancy
ADA criteria	Patients on anti-platelet drugs
1. HbA1C > 6.5% or	Pregnant females
2. Fasting plasma glucose > 126mg/dl (no caloric intake for at least 8 hours) or	Patients with any diagnosed malignancy
3. 2-hour plasma glucose >200 mg/dl (During a 75 gm oral glucose tolerance test) or	
4. Random plasma glucose > 200 mg/dl (plus classic symptoms of hyperglycemia including polyuria, polydipsia, polyphagia, and weight loss)	

Methods

Using a dry, disposable syringe and needle along with little stasis, all blood samples were taken from the antecubital vein. The specimens were then marked with the patients' ages, sex, and identification numbers. Ethylenediamine tetraacetic acid (EDTA) samples (for HbA1c) were kept at room temperature until processed within four hours of collection. HbA1c was estimated using the high-performance liquid chromatography (HPLC) method. An automated cell counter provided Hb values along with the platelet count and platelet indices (MPV, P-LCR, and PDW).

The methods used for the assessment of microvascular complications were:

Diabetic nephropathy: First morning sample for urinary albumin-creatinine ratio (UACR). Subjects with UACR of >30 mg/g were considered to have diabetic nephropathy.

Diabetic retinopathy: Fundus examination using fundoscopy.

Diabetic neuropathy: Clinical neurological examination (including sensory system, motor system, and deep tendon reflexes).

A standardized study proforma was used to examine each study participant. The length of diabetes, prior history of stroke, ischemic heart disease, and hypertension were all noted in the full history that was gathered. The individuals got a thorough clinical assessment. Diabetics were evaluated with special reference to microvascular complications.

Statistical analysis

A maximum 5% risk, a minimum of 85% power, and a 5% significance threshold (significant at a 95% confidence interval) were taken into consideration while calculating the sample size. A Microsoft Excel spreadsheet (Microsoft Corporation, Redmond, WA, US) was used to store the raw data, which was then analyzed using Statistical Package for the Social Sciences (SPSS) version 22.00 (IBM Corp., Armonk, NY, US). The mean and standard deviation for continuous data were displayed. 

Categorical data were expressed as percentages. Numerical variables were normally distributed and were compared using the chi-square test for non-parametric data, the independent ‘t’ test, and analysis of variance (ANOVA) Tuckey’s posthoc test for parametric data. The p-value was then determined to evaluate the level of significance. The results were analyzed and compared to previous studies to draw relevant conclusions.

## Results

The mean age was 56.9610±8.99 years among the cases and 55.102±11.36 years in the control group. Out of 100 cases, 57% (n=57) were females and 43% (n=43) were males and out of 100 controls, 48% (n=48) were females and 52% (n=52) were males. Sixty-three percent (63%; n=63) of the cases have a duration of diabetes between 5 and 10 years, 30% (n=30) have a duration of diabetes between 10 and 15 years, and 7% (n=7) of the patients have a duration of diabetes >15 years.

Among the cases, 43.8% (n=25) of the females had a waist-hip ratio of <0.85, and 56.2% (n=32) had a waist-hip ratio of >0.85 with a mean of 0.854±0.131 while among the controls, 64.5% (n=31) of the females had a waist-hip ratio of <0.85 and 35.5% (n=17) had a waist-hip ratio of >0.85 with a mean of 0.805±0.125. The p-value was 0.04 4, which is statistically significant. Among the cases, 48.8% (n=21) of the males had a waist-hip ratio of <0.90 and 51.2% (n=22) had a waist-hip ratio of >0.90 with a mean of 0.853±0.140, while amongst the controls, 82.6% (n=43) of the males had a waist-hip ratio of <0.90 and 17.4% (n=9) had a waist-hip ratio of >0.90 with a mean of 0.785±0.102. The p-value was 0.002, which is statistically significant. The mean body mass index (BMI) in the cases group was 28.212±3.56 kg/m^2^ while in the control group, the mean BMI was 21.241±2.092 kg/m^2^ (p=0.001). The mean glycated hemoglobin (HbA1c) among the cases was 9.014±1.957%.

The mean MPV among the cases was 12.089±1.450 fL and among the controls was 9.464±1.424 fL. In the cases group, 45% (n=45) of the patients had an MPV <11.7 fL, and 55% (n=55) had an MPV >11.7 fL. Ninety-five percent (95%; n=95) of the controls had an MPV <11.7 fL and 5% (n=5) had an MPV >11.7 fL. The p-value is 0.001, which is statistically significant. The mean PDW among the cases was 16.868±2.352 fL, and among the controls, it was 12.753±10.559 fL (p=0.001).

The mean P-LCR among the cases was 34.971±8.056 % and among the controls was 34.971±8.056 (p=0.001). The mean MPV in the patients with diabetic retinopathy was 13.1051±1.34492 fL and in patients without diabetic retinopathy, it was 10.0318±1.81039 fL, with a p-value of 0.001, as shown in Table 3.

**Table 3:** The association between diabetic retinopathy and mean platelet volume (MPV)

Diabetic retinopathy	MPV (fL)
	Mean± SD
Present	13.1051±1.34492
Absent	10.0318±1.81039
Average MPV	12.0890±1.45018
p-value	0.001

The mean PDW in the patients with diabetic retinopathy was 17.9682±2.52669 fL and in patients without diabetic retinopathy, it was 16.5577±2.22667 fL with a p-value of 0.012, which was statistically significant, as shown in Table 4.

**Table 4:** The association between diabetic retinopathy and platelet distribution width (PDW)

Diabetic retinopathy	PDW (fL)
	Mean± SD
Present	17.9682±2.52669
Absent	16.5577±2.22667
Average PDW	16.8680±2.35297
p-value	0.012

The mean P-LCR in the patients with diabetic retinopathy was 35.373±8.0629%, and in patients without diabetic retinopathy, it was 31.863±7.0714% with a p-value of 0.020, which was statistically significant, as shown in Table 5.

**Table 5:** The association between diabetic retinopathy and platelet large cell ratio (P-LCR)

Diabetic retinopathy	P-LCR (%)
	Mean± SD
Present	35.373±8.0629
Absent	31.863±7.0714
Average P-LCR	34.971±8.0560
p-value	0.020

Similarly, it was found that MPV, PDW, and P-LCR were significantly higher in patients with diabetic neuropathy and diabetic nephropathy (measured by UACR).

**Figure 1:**
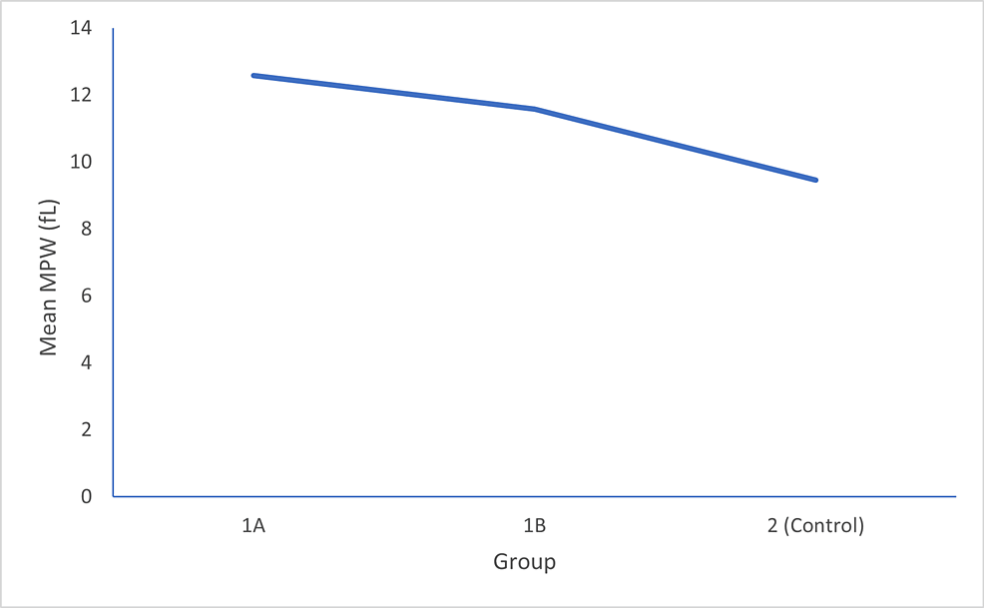
A comparison of mean platelet volume (MPV) in all three groups

In Group 1A, the mean MPV was 12.5960±0.95660 fL. In Group 1B, the mean MPV was 11.5820±1.67609 fL, and in Group 2, the mean MPV was 9.4641±1.42429 fL. The p-value between Group 1A and 1B was 0.002, between Group 1B and 2 was 0.001, and between Group 1A and 2 was 0.001, which was statistically significant, as shown in Figure 1.

**Figure 2:**
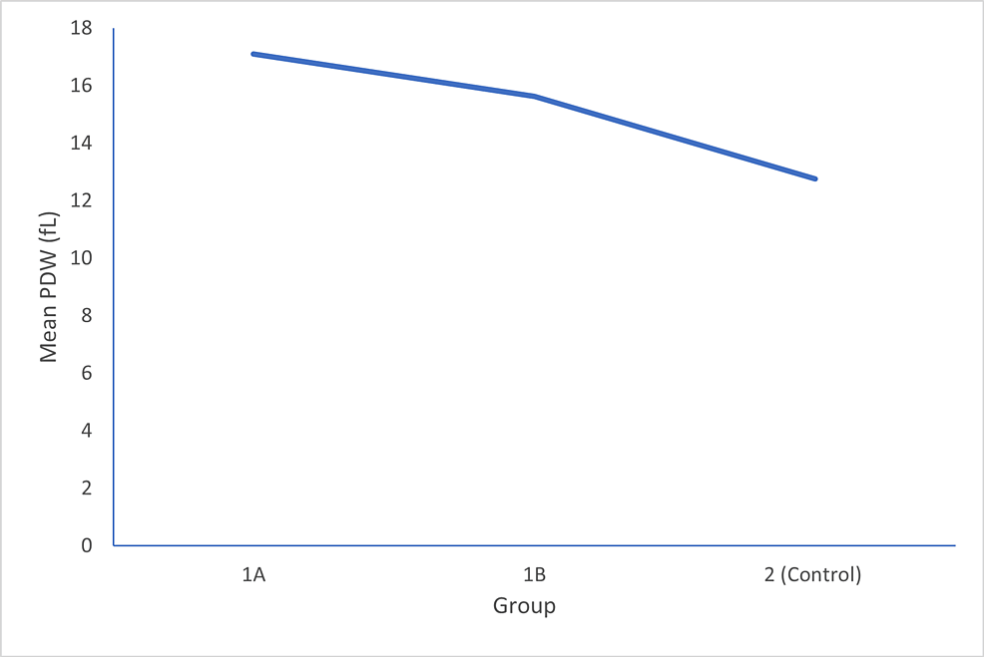
A comparison of platelet distribution width (PDW) in all three groups

In Group 1A, the mean PDW was 17.1140±2.58228 fL. In Group 1B, the mean PDW was 15.6220±2.10532 fL, and in Group 2, the mean PDW was 12.7539±10.55966 fL. The p-value between Group 1A and 1B was 0.002, between Group 1B and 2 was 0.001, and between Group 1A and 2 was 0.001, which was statistically significant, as shown in Figure 2.

**Figure 3:**
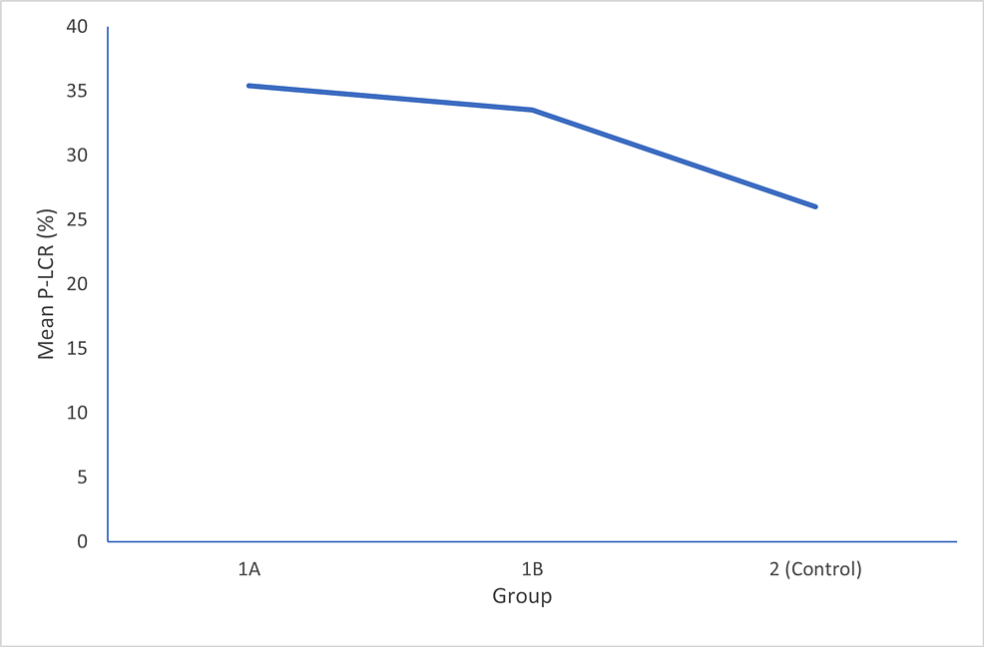
A comparison of mean platelet large cell ratio (P-LCR) in all three groups

In Group 1A, the mean P-LCR was 35.408±3.5490%. In Group 1B, the mean P-LCR was 33.542±4.8694%, and in Group 2, the mean P-LCR was 26.030±7.0049%. The p-value between Group 1A and 1B is 0.031, between Group 1B and 2 is 0.001, and between Group 1A and 2 is 0.001, which was statistically significant, as shown in Figure 3.

## Discussion

In our study, the mean age of cases was 56.961±8.99 years and that of controls was 55.102±11.36 years. Among the 100 cases, there was a female preponderance, with 57% females and 43% males, and out of the 100 controls, 48% were females and 52% were males. A study on platelet indices in diabetes mellitus had a mean age of diabetic patients 51.75±11.4 years and 48·12 years±13·69 years in control subjects. There was an incidental female predominance in both groups (male/female: 1∶2·3 in controls and 1∶2·88 in diabetic patients) as seen in our study. Among the cases, 63% had a duration of diabetes between 5 and 10 years, 30% had a diabetes duration between 10 and 15 years, and 7% had a duration of 15 years or more [11].

In another study, the conclusion that large sex-ratio differences are observed with a higher incidence among women as the most prominent risk factor of diabetes, which is obesity, is more common in women. Psychosocial stress appears to have a greater impact on women rather than on men. Moreover, women have greater increases in cardiovascular risk, myocardial infarction, and stroke mortality than men [12].

In the present study, the average HbA1c was 9.014±1.957% and the fasting plasma glucose (FPG) among the cases was 235.243±82.546 mg/dl. The lipid profile was deranged amongst the cases as compared to the controls. The mean serum cholesterol in the cases was 160.982±52.088 mg/dl while in controls, it was 130.943±50.412 mg/dl with a p-value of 0.001, which is statistically significant. Similarly, there was a significantly higher value of serum triglyceride and serum low-density lipoprotein (LDL) and a significantly lower value of serum high-density lipoprotein (HDL) among the cases in comparison to the controls with a p-value of 0.001, 0.001, and 0.011, respectively.

Another study concluded that dyslipidemia in patients with type 2 diabetes is very common. Lipid abnormalities observed in these patients play a central role in the development of atherosclerosis. These abnormalities are not only quantitative but also qualitative and kinetic in nature. Many of the lipid abnormalities observed in patients with type 2 diabetes exist before the onset of diabetes as part of the insulin-resistant metabolic syndrome, which is characterized by the accumulation of triacylglycerol-rich lipoproteins (hypertriglyceridemia) and small, dense LDL particles with reduced HDL-cholesterol levels in plasma. The primary (characteristic) quantitative abnormalities are hypertriglyceridemia, increased production of triacylglycerol-rich lipoproteins, a reduction in the rate of catabolism of triacylglycerol-rich lipoproteins, and decreased HDL-cholesterol levels secondary to an increased rate of HDL catabolism [13].

Platelet indices

The average mean platelet volume (MPV) in the diabetics was 12.089±1.450 fL as compared to the controls where it was 9.464±1.424 fL with a statistically significant difference and a p-value of 0.001. This is comparable to MPV values in a study where the mean MPV in diabetics was 11.32±1.72 fL while in the controls the mean value was 6.56±1.51 fL. Similarly, platelet distribution width (PDW) among the cases was 16.868±2.352 fL while in controls it was 12.753±10.559 fL with a p-value of 0.001. The mean PDW among the diabetics in the study quoted above was 15.57±3.23 fL which was significantly higher than that among the controls [5]. In our study, the mean P-LCR was 34.975±8.056% among the cases in comparison to the mean P-LCR among the controls, which was 26.031±7.004%. The mean P-LCR was 43.15±10.68% with a statistically significant difference from the non-diabetics with a p-value of 0.001. It was also observed that the MPV, PDW, and P-LCR were significantly higher in diabetic individuals with microvascular complications as compared to those without complications [5].

The mean MPV in diabetics with complications was 12.5960±0.95660 fL and in those without complications was 11.5820±1.67609 fL with a p-value of 0.002. Similar results were obtained in cases of PDW and P-LCR. The mean PDW in diabetics with complications was 17.1140±2.58228 fL and without complications was 15.6220±2.10532 fL with a significant p-value of 0.002. The mean P-LCR in diabetics with microvascular complications was 35.408±3.5490% and without complications was 33.542±4.8694% with a p-value of 0.031. In the aforementioned study, it was found that these platelet indices have a higher value in diabetics with microvascular complications than those without complications with a mean MPV, PDW, and P-LCR being 12.35±1.50 fL, 15.65±3.01 fL, and 47.4±9.79%, respectively, in patients with complications. It had a statistically significant difference in the values of MPV, PDW, and P-LCR from the patients without complications with p-values of 0.0001, 0.0001, and 0.0001, respectively [14].

In our study, it was found that there was a statistically significant association between increasing HbA1c levels and MPV. The mean MPV in the patients with HbA1c <7.5% was 10.9440±1.88505 fL, in patients with HbA1c between 7.5-10%, it was 11.0527±1.36366 fL, and in patients with HbA1c >10%, it was 12.3700±1.03420 fL with a p-value of 0.031, which is statistically significant and supported by various studies. In a study involving 100 diabetics, who were divided into Group A (HbA1c<7%) and Group B (HbA1c>7%), and compared to 50 healthy controls (Group C), MPV was compared among them. In Group A, mean MPV was maximum in Group B (13.35±1.27 fL), followed by Group A (10.77±0.77 fL) and Group C (9.09±.85 fL) with a p-value of 0.05 [15]. Similar results were found in the study in which higher MPV values were found in patients with higher HbA1c with a p-value of <0.0001 [16].

However, the association between HbA1c and PDW & HbA1c and P-LCR showed an increase in the absolute values of PDW and P-LCR with higher values of HbA1c, but the relationship was not statistically significant. These findings are contrary to the findings of the study where a statistically significant association between increasing values of HbA1c and PDW and P-LCR with p-values of 0.005 and 0.0001, respectively [5].

In the present study, the mean MPV in the patients having normal BMI (18.50-22.90 kg/m^2^) was 12.0286±1.187 fL, in patients who were overweight (23.00-24.90 kg/m^2^), it was 12.4273±1.127 fL, in pre-obese (25.00-29.9 kg/m^2^) patients, it was 12.983±1.227 fL, and in patients who were obese (>30.00 kg/m^2^), it was 13.952±1.224fL. The p-value is 0.042, which is statistically significant. In a study, similar statistical significance was found between MPV and BMI with a p-value of 0.02. The mean PDW in the patients having normal BMI (18.50-22.90 kg/m^2^) was 16.1000±2.407 fL, in patients who are overweight (23.00-24.90 kg/m^2^), it was 16.4091±2.653 fL, in patients who are pre-obese (25.00-29.90 kg/m^2^), it was 17.1073±2.458 kg/m^2^ and in patients who are obese (>30.00 kg/m^2^), it was 18.6667±2.068 fL. The p-value is 0.038, which is statistically significant. The mean P-LCR in the patients having normal BMI (18.50-22.90 kg/m2) was 31.843±9.708%, in patients who are overweight (23.00-24.90 kg/m^2^), it was 34.695±7.636%, in pre-obese (25.0-29.9 kg/m^_2_^) patients, it was 35.793±8.677%, and in patients who are obese (>30.00 kg/m^2^), it was 36.364±8.001%. There is an increase in the values of P-LCR with increasing BMI but the p-value is 0.637, which is not statistically significant [16,17].

Diabetic microvascular complications

The mean MPV, PDW, and P-LCR in the patients with diabetic retinopathy were 13.1051±1.34492 fL, 17.9682±2.52669 fL, and 35.373±8.0629%, respectively, which is higher than those without diabetic retinopathy with a p-value of 0.001, 0.012, and 0.020, respectively. This is in accordance with the results of studies, where similar conclusions were drawn [17-19]. The mean MPV, PDW, and P-LCR were significantly higher in patients with diabetic neuropathy than those without diabetic neuropathy as also seen in another study. It was observed that MPV, PDW, and P-LCR showed a positive correlation with diabetic neuropathy, which was significant (p = 0.0001, p = 0.023, p = 0.0001). In our study, a statistically significant positive association of the three platelet indices was found with diabetic nephropathy (UACR >30 mg/g) [5]. However, other studies have shown that only MPV and PDW were significantly higher in diabetic nephropathy [19].

Limitations of the study

The sample size of 200 (100 patients and 100 controls) could only provide results at a maximum 5% risk, a minimum of 85% power, and a 5% significance threshold (significant at a 95% confidence interval, so a large-scale sample size would have much better results and the group of the patients constituted a part of homogeneous population, so the results may vary for heterogeneous populations, which we will consider as the limitation of the present study.

## Conclusions

Based on the findings of the present study, there is a statistical correlation between type 2 diabetes and variations in platelet indices, resulting in the associated microvascular complications. Higher MPV, PDW, and P-LCR values suggest that these parameters are more reliable predictors of early vascular complications in individuals with type 2 diabetes mellitus and can be utilized as an easy-to-use, low-cost method. They are a readily available, economical, practical, noninvasive, and simple-to-understand approach for assessing platelet dysfunction, which in turn helps anticipate the existence of microvascular complications. We were also able to determine that platelet indices are a reliable indicator of the development of microvascular complications in type 2 diabetic individuals.
